# Prevalence, classification and dental treatment requirements of dens invaginatus by cone-beam computed tomography

**DOI:** 10.7717/peerj.14450

**Published:** 2022-12-05

**Authors:** Turgut Yagmur Yalcin, Kıvanç Bektaş Kayhan, Ayca Yilmaz, Sevde Göksel, İlknur Ozcan, Dilek Helvacioglu Yigit

**Affiliations:** 1 Department of Endodontics, Faculty of Dentistry, Istanbul University, Istanbul, Turkey; 2 Department of Oral and Maxillofacial Surgery, Faculty of Dentistry, Istanbul University, Istanbul, Turkey; 3 Department of Oral and Maxillofacial Radiology, Faculty of Dentistry, Istanbul University, Istanbul, Turkey; 4 QU Health, College of Dental Medicine, Qatar University, Doha, Qatar

**Keywords:** Cone-beam computed tomography, Dens invaginatus, Dental anomalies, Endodontic treatment, Prevalence

## Abstract

**Background:**

This study aimed the evaluation of the prevalence, characteristics, types of dens invaginatus (DI) and co-observed dental anomalies to understand dental treatment requirements in anterior teeth that are susceptible to developmental anomalies by using cone-beam computed tomography (CBCT).

**Methods:**

In this retrospective study, the anterior teeth of 958 patients were evaluated by using CBCT for the presence of DI. The demographic features, types of DI and treatment requirements were also recorded. The association between sex and the presence of DI was evaluated using chi-squared test.

**Results:**

Seventy-three DI anomalies were detected in the anterior teeth of 49 patients (18 females, 31 males). The frequency of DI was 5.11% and the most frequently involved teeth were lateral (57.53%). Forty-six teeth were classified as Type I (63.01%), 24 as Type II (32.87%), and three as Type III (4.10%). Apical pathosis was found to be 20.54% in all DIs detected and accounted for all Type III and one-third of Type II.

**Conclusions:**

CBCT imaging can be effective in the detection of dental anomalies such as DI and planning for root canal therapy and surgical treatments. Prophylactic interventions might be possible to prevent apical pathosis with the data obtained from CBCT images.

## Introduction

Dens invaginatus (DI) is a dental anomaly that is thought to result from the invagination of the enamel organ towards the dental papillae during tooth development (Hulsmann, 1997). It was first described in human teeth by Socrates in 1856 (Hulsmann, 1997). This anomaly can be seen in permanent, deciduous, and supernumerary teeth (Jimenez-Rubio et al., 1997). Any of the teeth in the maxillary and mandibular arch may be affected by DI, but the maxillary lateral incisors are most commonly affected (Schwartz & Schindler, 1996; Sousa Neto et al., 1992; Altinbulak & Ergul, 1993; Kumar et al., 2014; Cakici et al., 2010). The frequency of permanent teeth affected by DI is variable, ranging from 0.04%–10% (Goncalves et al., 2002; Alani & Bishop, 2008; Rotstein et al., 1987). DI is mostly observed in single invagination form, while double (NuNu Lwin, Phyo Kyaw & Wai Yan Myint Thu, 2017; Zoya et al., 2015) and triple forms (Chhina et al., 2017; Serrano, 1991) have rarely been reported. The bilateral occurrence of DI is common, and its frequency was found to be between 24.2 and 82% (Moss, 1967). The presumed etiology of DI involves both genetic and environmental factors (Hulsmann, 1997; Zhu et al., 2017; Gunduz et al., 2013). Generally, no association between prevalence of DI and gender has been reported in the literature (Cakici et al., 2010; Hegde et al., 2022; Alkadi et al., 2021; Mabrouk, Berrezouga & Frih, 2021; Kirzioglu & Ceyhan, 2009; Hamasha & Alomari, 2004; Ceyhanli et al., 2015).

A wide variety of anatomical formations was observed based on the amount of invagination. This has led to the formation of different classifications. The main types of DI are coronal and radicular DI. The coronal type is more common, and the most used classification on coronal type was proposed by Oehler’s classification (Kumar et al., 2014; Alani & Bishop, 2008). This classification is described as follows:

Type 1: Enamel-lined invagination confined to the coronal part of the tooth not extending beyond the cementoenamel junction.

Type 2: Extension of the invagination into the root, beyond the cementoenamel junction, ending as a blind sac; the invagination may or may not communicate with the pulp.

Type 3a: The invagination extends through the root and communicates with the periodontal ligament space at the lateral side through a pseudo-foramen without any communication with the pulp tissue.

Type 3b: The invagination extending through the root, communicates with the periodontal ligament at the apex. Generally, no sign of communication with the pulp tissue has been noted. The thin layer of dentine and enamel between the pulp tissue and the invagination causes the entry of irritants into the root canal system. Thus, invagination is considered a predisposing factor for caries. Furthermore, pulp necrosis might occur related to incomplete enamel-lining in some cases (Hulsmann, 1997).

Microorganisms and their products may aggravate infection and lead to necrosis of the pulp (Sousa Neto et al., 1992). Therefore, the extension of invagination, type of DI and the condition of pulp changes the treatment options and course of treatment. The aim of the treatment should be maintaining the pulp vitality and preserving the tooth structure by minimum invasive method (Zhu et al., 2017). The complex anatomy of DI makes it difficult and unpredictable to treat such teeth. Thus, several treatment modalities have been described for different types of DI, ranging from conservative restorative procedures to nonsurgical root canal therapy, surgery, or extraction (Kirzioğlu & Ceyhan, 2009).

Cone-beam computed tomography (CBCT) is a well-established three-dimensional (3D) imaging tool, which has several advantages compared to 2D radiographs including superior diagnostic ability in the detection of DI and assessment of its type. The location of the extension of invagination and the course of penetration into the affected tooth can be visualized in a more detailed way by the use of CBCT. Furthermore, assessment of DI’s association with apical lesion may aid in the selection of treatment approaches for DI cases. CBCT provides information that is more accurate than periapical or panoramic radiographs regarding the periapical lesions (Leonardi Dutra et al., 2016). For these reasons, it may be useful to have information on the types of DI and their association with periapical lesion prior to treatment planning, especially by CBCT instead of 2D radiographs in complicated cases (Capar et al., 2015; Rozylo, Rozylo-Kalinowska & Piskorz, 2018). To our knowledge, there are limited number of studies that have investigated DI using CBCT (Alkadi et al., 2021; Mabrouk, Berrezouga & Frih, 2021; Capar et al., 2015).

DI is not an uncommon clinical finding in permanent dentition; thus, it may be easily overlooked in the absence of clinical signs (Alani & Bishop, 2008). This is concerning as the presence of any type of DI may increase the risk of caries, pulpal pathosis, internal resorption of the involved tooth, periodontal inflammation, and moreover, complicates the endodontic treatment (Schwartz & Schindler, 1996; Sousa Neto et al., 1992; Altinbulak & Ergul, 1993; Alani & Bishop, 2008). The maxillary anterior region has special importance in facial and dental esthetic, therefore tooth loss and malformed structures could affect the appearance as well as the quality of life of an affected individual. This region has particular importance when it comes to dental anomalies and stands at the top of the list as being the most affected region of mouth regarding dental anomalies including DIs (Kfir et al., 2020).

To date, the prevalence of DI in different populations was investigated many times, due to the diversity of techniques used, there were no exact type-specific prevalence data and the need for prophylactic treatment were not put forth. In this study, the prevalence and type of DI, the presence of apical pathosis and prophylactic treatments were specially examined.

## Materials & Methods

### Ethical approval

This retrospective study was performed using the CBCT images of 2,807 patients who applied to the Faculty of Dentistry of the Department of Oral and Maxillofacial Radiology of Istanbul University between December 2015 and May 2018. This study was conducted according to the principles of the Declaration of Helsinki and approved by IRB committee of Istanbul University Faculty of Dentistry (Ref. 2018/33). Information on orodental, medical (genetic disorders, systemic disease), and demographic characteristics of patients were obtained from clinical records.

### Selection criteria

The CBCT (Soredex SCANORA^®^3Dx; Soredex, Tuusula, Finland) was used with a tube voltage of 90 kV. The CBCT images were evaluated using a computer program (Ondemand 3D Project Viewer; Cybermed Inc., Tustin, CA, USA) on a monitor (Dell 24 UltraSharp monitor-U2415) in a dimly lit room. Only patients with a full set of maxillary and mandibular anterior teeth and complete clinical records were included in the study. Low-quality images, such as those with scattering or insufficient accuracy of bone borders, were excluded.

### CBCT image evaluation and DI detection

**Figure 1:**
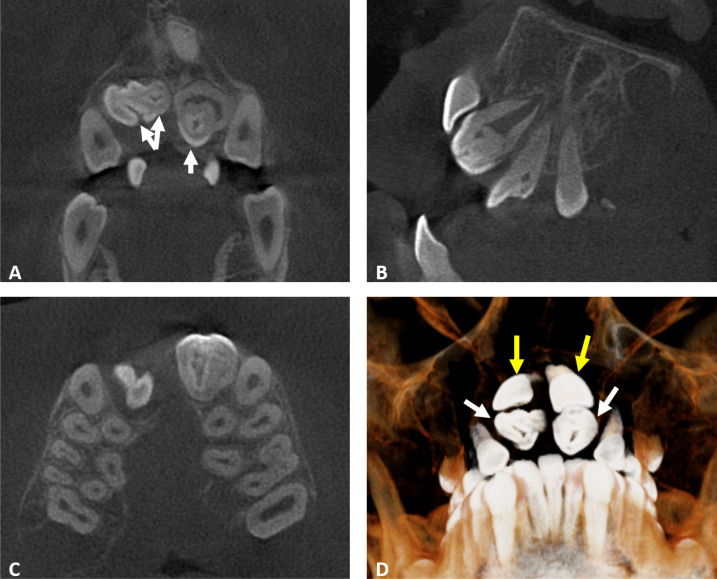
Coronal, sagittal, axial, and cross-sectional CBCT images that are examined. (A) Coronal CBCT section showing the presence of double invaginatus in 11 and enamel lined invaginatus of the maxillary lateral incisors (white arrows). (B) Sagittal section revealing the presence of dens invaginatus Oehlers Type 1 in the maxillary lateral incisors. (C) Axial section showing the unilateral cleft palate on the right side. (D) Three-dimensional reconstruction showing the altered morphology of lateral incisors (white arrows) and the impacted central incisors (yellow arrows).

**Figure 2:**
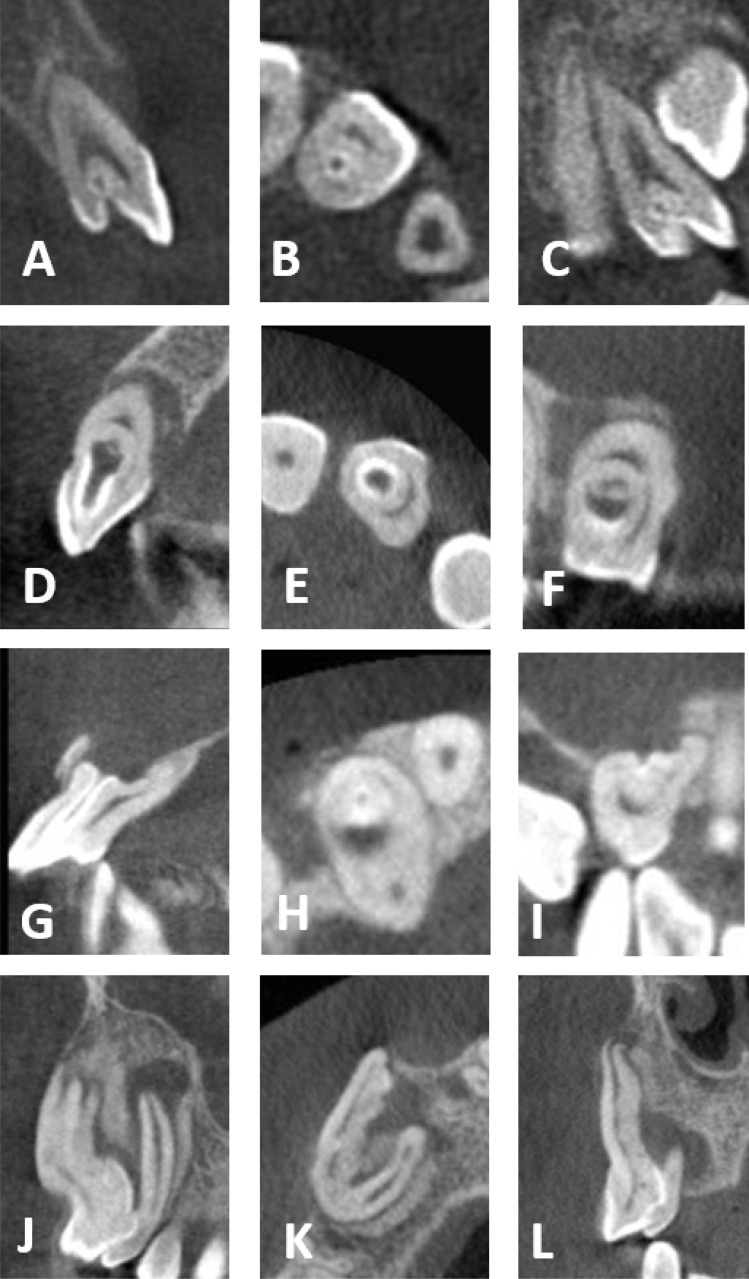
CBCT images of the four DI types. (A–C) Maxillary lateral incisor with DI type I. (D–F) Maxillary lateral incisor with DI type II. (G–I) Maxillary lateral incisor with DI type III A. (J–L) Maxillary canine with DI type III B. (A, D, G, J) Sagittal sections. (B, E, H, K) Axial sections. (C, F, I, L) Coronal sections.

Anterior region teeth (including supernumerary and deciduous teeth) in 958 patients who met the inclusion criteria were examined for the presence of DI. DI was defined as an in-folding of a radiopaque ribbon-like structure equal in density to the enamel extending from the cingulum into the root canal. Coronal, sagittal, axial, and cross-sectional images were examined from various angles by an endodontist, and an oral radiologist both of whom had twenty years’ experience (Figs. 1 and 2). An oral and maxillofacial radiologist with 30 years’ experience was consulted for decisive evaluation if a consensus had not yet been reached as to the presence of DI.

### DI assessment

After DI cases were detected, the patients were evaluated for age, sex, frequency, double and triple invagination frequency, the potentially disproportionate rate of DI occurrence in a particular group of teeth, bilateral occurrence frequency, side, types of single and double forms of DI and possible detection in panoramic films. The presence of syndromes, systemic diseases and dental or facial trauma histories, and dental anomalies of the DI patients were also investigated. In patients with three or more cases of DI, if a pair of teeth with DI was detected bilaterally, the patient was added to the bilateral occurrence group. On the classification of the sides of occurrence of DI, three main groups (right, left and midline) emerged. Mesiodens teeth with DI were included in the midline group. Referral reasons, the presence of syndromes and systemic diseases, and dental anomalies in the DI patients were recorded. The existence of any dental anomaly within the examined region was also recorded. The crown shapes of teeth with DI were also examined using CBCT data and dental records. In case of double invagination existence, the classification of the deeper invagination was taken into consideration

After having assessed the different types of DI, the authors evaluated the distribution of different types of DI according to patients, detected side, presence of carries, open apex and apical pathology, and types of double invagination detected. In addition, clinical notes about treatment were recorded.

### Statistical analysis

Statistical analyses were conducted using IBM SPSS Statistics 22 (IBM SPSS, Armonk, NY, USA), inter-examiner reliability between the two observers was calculated using the Cohen’s kappa test. According to the Cohen’s kappa test, the inter-examiner agreement was high between the two assessments of the observers, with a value of *k* = 0.934, cronbach alpha (*α*) = 0.954, intra-examiner value = 0.948−0.959, *p* < .001. Statistical evaluation of the presence of DI related to age and gender was performed using the Pearson correlation and the chi-squared test.

## Results

In this study, patients’ ages ranged from 9 to 61 years (a mean age of 24.82 years). Seventy-three DI anomalies were detected in the anterior teeth of 49 patients (18 females, 31 males) (Table 1). DI anomalies were not related to gender (*χ*^2^ (1, *N* = 958) = 0.374, *p* = 0.541) or age (*r* (47) = −.048, *p* > .05). A total of 73 cases of DI were included, 10 double invagination teeth were observed among two females, and seven males and one male patient had two double invagination teeth. Panoramic radiographs were unsuccessful in detecting DI in 54 teeth of 35 patients in this study which corresponded to 73.97% of total DI detected in CBCT images and 71.42% of the patients. The 39 of 46 Type I, 14 of 24 Type II and 1 of 3 Type III were not detectable in panoramic radiographs.

**Table 1:** Descriptive data. The prevalence, characteristics, and types of dens invaginatus (DI).

Parameters		Frequency of distribution	
		*n*	**%**
Number of patients assessed		958	
Patients with DI (overall patient prevalance)		49	5.11%
Gender	Female	31	63.27%
	Male	18	36.73%
Number of teeth assessed		11,774	
Anterior teeth with DI (overall teeth prevalance)		73	0.62%
Unilateral		30	
Bilateral		24	
Mesiodens		19	
Tooth Type	Central	5	6.86%
	Lateral	43	58.9%
	Canine	3	4.11%
	Supernumerary	22	30.14%
Type of DI	Type I	43	58.9%
	Type II	17	23.29%
	Type III	3	4.11%
	Double Type I	3	4.11%
	Double Type II	7	9.59%
Pal Status	Absent	58	79%
	Present	15	21%
Crown Morphology	Normal	36	49.31%
	Barrel	12	16.44%
	Amorphous	23	31.51%
	Conical	2	2.74%

**Notes.**

DI Dens Invaginatus PAL Periapical Lesion

The frequency of DI per patient was 5.11%, doubled form 0.94% in the anterior region teeth. Among DI inspected teeth, 97.26% were in the maxillary arch. Only two DI cases (one canine and one lateral incisor teeth) were observed in the mandibular teeth. The most common teeth detected with DI were maxillary lateral incisors teeth (*n* = 42), followed by supernumerary (*n* = 22) and maxillary incisors (*n* = 5), respectively. 113 supernumerary teeth (93 impacted position) were detected, and 22 of them (19 impacted) were affected by DI, 8 of which had double invagination.

DI was not detected in deciduous teeth among 165 teeth inspected. DI was found to be unilateral in 75.51% of cases and bilateral in 24.48% of cases. 19 of the supernumerary teeth affected by DI were mesiodens. 41.09% (*n* = 30) of the affected teeth were found on the right side, 32.87%(*n* = 24) on the left and 26.02% (*n* = 19) of DI were found in the midline.

There were three patients with systemic diseases (one hypertension, one epilepsy, and one patient who had rheumatoid arthritis and scoliosis); associations of DI with systemic diseases were not detected. In syndromic patients; DI occurred (five in anterior, one posterior mandibulary instances) in a patient with an *α*1-antitrypsin deficiency (ATD) genetic disorder, in four patients with cleft lip/palate five DI teeth was found, one of whom had two DI affected teeth. Another patient with cleidocranial dysostosis had 11 impacted teeth among three also had DI.

When the referral reasons for CBCT were considered in the present retrospective study, the foremost reason for referral was having impacted teeth (24.4%), supernumerary teeth (18.4%), DI (12.2%), cysts (12.2%), dental anomaly other than DI (8.2%), followed by radioopaque lesions, cleft lip and palate, compound odontoma, dental trauma, extraoral sinus tract (4% each), and hypercementosis, odontogenic tumor (2% each) respectively.

Almost half of the teeth with DI had normal crown morphology (*n* = 36, 49.31%). DI teeth with abnormal crown morphology were amorphous (*n* = 23, 31.5%), barrel-shaped (*n* = 12, 16.43%), or conical (*n* = 2, 2.73%).

According to Oehler’s classification, present study consisted of only coronal DI’s, and any radicular DI has not been detected. Among classified types, 46 teeth as being Type I (63.01%), 24 as Type II (32.87%), and 3 as Type III (4.1%) have been detected. All Type III teeth, 33.3% of Type II teeth, and 8.69% of Type I teeth showed signs of apical pathosis at the time of referral (Table 2). The different types of DI occurrences in the same patients were evaluated. Type I and II were concurrently detected in eight patients. When the double invaginations were examined, it was found that the depths of the two invagination lines were very close to each other in all teeth. Demographic data, classification of DI according to tooth number, Oehler’s classification types, and the related clinical information of each DI were given in Table 1.

## Discussion

Panoramic and/or periapical radiographs are used retrospectively in full-mouth survey studies on DI frequency (Cakici et al., 2010; Gunduz et al., 2013; Colak et al., 2012). These imaging methods are routine procedures in dental examinations and accordingly, a large amount of data can be obtained. The CBCT imaging technique is not a routine procedure due to the doses of radiation it incurs on healthy patients. CBCT is preferred only when the benefit outweighs the risks and is generally used with a smaller field of view (FOV) values in order to minimize the patient’s exposure to radiation (anon, 2011). Therefore, a comprehensive survey study of the entire mouth with CBCT is not feasible, which is one of the reasons that the present study only focused on the teeth in the anterior region.

**Table 2:** Distrubition of DI teeth with PAL according to tooth type and DI type.

	Tooth type	DI type	*n*
Total DI teeth with PAL	Central =1	Type I	
		Type II	1
		Type III	
		Double Type I	
		Double Type II	
	Lateral =12	Type I	4
		Type II	7
		Type III	1
		Double Type I	
		Double Type II	
	Canine=2	Type I	
		Type II	
		Type III	2
		Double Type I	
		Double Type II	

**Notes.**

DI Dens Invaginatus PAL Periapical Lesion

According to the methodology used in previous studies, the frequency of DI was reported to be ranging between 0.25% (Poyton & Morgan, 1966) and 38.5% (Miyoshi et al., 1971) and in Turkish subpopulations varying between 1.3–12% (Cakici et al., 2010; Gunduz et al., 2013; Kirzioğlu & Ceyhan, 2009; Capar et al., 2015). There were a few differences between the studies, including different imaging systems being used, DI frequencies being calculated either per patient or per tooth, and the evaluation of only lateral incisors in some studies (Alani & Bishop, 2008). The different methodologies used in these studies thus make the comparison difficult. In studies using periapical and/or panoramic radiographs, the prevalence of DI was reported to be 1.3% (Cakici et al., 2010), 2.5% (Gunduz et al., 2013), and 2.95% (Colak et al., 2012) in Turkish subpopulations. In this study, the DI prevalence in CBCT images is reported to be 5.11%, and the rate of detecting DI in panoramic radiographs of the same patients was 1.46% in parallel with other studies used panoramic radiographs. Ceyhanli et al. (2015) detected 5.9% DI, whereas Capar et al. (2015) detected 10.7% DI per patient, as in the present study, from CBCT images. Although there are disparities between DI detection rates in CBCT images, the authors speculate that the referral of patients with dental anomalies to CBCT, which allows for the easy detection of DI, explains the higher frequency of DI detection in all studies with CBCT compared with panoramic radiographs. The detection of DI and its images provided by CBCT leads to early interventions and prophylactic treatments with feasible results (Ceyhanli et al., 2014; Vier-Pelisser et al., 2012).

Also the frequency of bilateral occurrence of DI was investigated and found 24.48% in this study, 31.3% according to Capar et al. (2015), and 24.2% according to Rozylo, Rozylo-Kalinowska & Piskorz (2018). All three studies were reports of CBCT images.

A number of studies that used different methodologies have revealed that the most commonly DI affected teeth were maxillary lateral incisors (Cakici et al., 2010; Gunduz et al., 2013; Kirzioğlu & Ceyhan, 2009; Rozylo, Rozylo-Kalinowska & Piskorz, 2018). This was followed by maxillary central incisors, as per studies using conventional radiographic methods (Gunduz et al., 2013; Kirzioğlu & Ceyhan, 2009; Gallacher, Ali & Bhakta, 2016; McNamara, Garvey & Winter, 1998; Munir et al., 2011). In contrast, supernumerary teeth were ranked as second affected teeth in studies using CBCT (Capar et al., 2015; Rozylo, Rozylo-Kalinowska & Piskorz, 2018). When DI frequency for tooth types in the anterior region was evaluated, 19.46% of 113 supernumerary teeth and 2.19% of 1916 maxillary lateral teeth appeared to be affected by DI. According to tooth type classifications, supernumerary teeth were the most DI affected teeth in this study. Using CBCT, Rozylo, Rozylo-Kalinowska & Piskorz (2018) and Capar et al. (2015) found 29.3% and 9% of supernumerary teeth in all DI cases, respectively. However, since these studies did not reveal the number of supernumerary teeth not affected by DI, the results were not comparable. The authors also examined the studies using periapical and/or panoramic images and found that DI occurrences in supernumerary teeth were not reported (Cakici et al., 2010; Gunduz et al., 2013; Hamasha & Alomari, 2004; Kirzioğlu & Ceyhan, 2009; Colak et al., 2012). These results might have been affected by the facts that conventional radiographic methods are two-dimensional, supernumerary teeth are usually impacted (vertical or inverted positions) (Van Buggenhout & Bailleul-Forestier, 2008; Zhu et al., 1996; Tyrologou, Koch & Kurol, 2005), and sometimes impacted supernumeraries were extracted to facilitate the spontaneous emergence of permanent teeth (Van Buggenhout & Bailleul-Forestier, 2008; Güngör et al., 2005).

While still unclear, there are many theories that attempt to explain the etiology of DI formation (Hulsmann, 1997). Segura, Hattab & Rios (2002) claimed that external forces applied during tooth germ development, such as those of adjacent tooth germs, could be an etiological factor of DI. It is also known that supernumerary teeth may displace and thwart the emergence of adjacent teeth due to the pressure that is applied (von Arx, 1992; Rajab & Hamdan, 2002; Liu, 1995). Therefore, the idea that the interaction of the adjacent and supernumerary teeth germs or impacted teeth could contribute to DI formation during tooth germ developmental stages is worth investigated. This interaction could affect both teeth (the supernumerary and the adjacent tooth). This study identified 38 impacted teeth adjacent to DI affected teeth, as well as 22 supernumerary teeth with DI anomaly. While these results could support Segura, Hattab & Rios’s (2002) theory, comprehensive studies need to be planned to evaluate tooth germ developmental stage times. In addition to this, a number of fusion cases between supernumerary and anterior teeth, characterized by the union of the two different types of teeth, have been reported (Krishnamurthy et al., 2018; Khan et al., 2015; Bulut & Pasaoglu, 2017; Ghaderi & Rafiee, 2016). These case reports could lend support to the idea that developmental stages are close to one another. Fusion between supernumerary and anterior teeth is the most frequent dental anomaly that co-occurs with DI, as seen in three cases in the present study.

Genetic factors are another possible etiological factor of DI (Hulsmann, 1997) and as such also warrant discussion. According to Kettunen et al.’s (2000) study on tooth morphogenesis, growth molecules regulate enamel organ forming. Therefore, the genetic inheritance of deficient growth factors could induce morphological anomalies (Ireland, Black & Scures, 1987; Pokala & Acs, 1994; Oncag, Gunbay & Parlar, 1995; Hibbert, 2005; Grahnen & Dens invaginatus, 1959). This theory is supported by case reports focused on siblings of DI affected patients who showed similar anomalies to those of their parents (Grahnen & Dens invaginatus, 1959; Sarraf-Shirazi, Rezaiefar & Forghani, 2010; Hosey & Bedi, 1996). Pokala & Acs (1994) reported a patient with a deficiency in chromosome 7g32 who presented a number of dental anomalies such as DI and hypodontia. DI has also been associated with other dental anomalies such as gemination, microdontia, macrodontia, absence of permanent tooth germs, taurodontism, supernumerary tooth, and dentinogenesis imperfecta (Van Buggenhout & Bailleul-Forestier, 2008). In addition, this study demonstrated a patient with an *α*1-antitrypsin deficiency genetic disorder who had six occurrences of DI, six taurodont, and one dens evaginatus dental anomaly. In addition, four cleft lip/palate patients had DI, one of whom had two DI affected teeth. Cleidocranial dysplasia patient with DI has been reported as well. Taken together, these results lend support to the genetically linked nature of DI.

Most often DI is detected by chance on the radiograph. Clinically, an unusual crown morphology or a deep foramen caecum may provide important hints, but affected teeth may also fail to show any clinical signs of malformation (Hulsmann, 1997). When the authors examined the tooth shapes in patients enrolled in this study, almost half of the teeth with DI had normal crown morphology. The crowns that had abnormal morphology demonstrated amorphous (31.5%), barrel-shaped (16.43%) and conical (2.73%) shapes. Rozylo, Rozylo-Kalinowska & Piskorz (2018) found that about one third of the teeth with DI had amorphous morphology similar with this study. Conversely, Kirzioğlu & Ceyhan (2009) detected 795 DI affected teeth, and on examining DI crown morphologies, only 5% of them showed abnormal crown morphology.

In general, DI could be classified as radicular and coronal type; since the incidence of radicular DI is extremely low, the term DI refers to coronal DI in general, as in this study (Munir et al., 2011). From the clinicians’ perspective, classifications have a special importance when the treatment outcomes are affected due to this scheme. Oehler’s classification has been used in several studies to determine the prevalence of DI; in this study, the authors preferred to use this classification due to its disseminated use in literature and high susceptibility of clinical relevance (Kirzioğlu & Ceyhan, 2009; Capar et al., 2015; Rozylo, Rozylo-Kalinowska & Piskorz, 2018).

Apical pathosis is a clinical problem, especially in the maxillary anterior region that could lead to unwanted aesthetic consequences. Early detection of DI and prophylactic practices would bring out the least number of apical pathosis and consequencing results such as extractions, surgical removal of related teeth, and cysts. Cakici et al. (2010) and Capar et al. (2015) detected apical pathosis in DI Type III while Capar et al. (2015) detected apical pathosis in 25% of DI Type II. Gunduz et al. (2013) observed apical pathosis in 87.5% of all Type III teeth. According to this study, apical pathology was found to be 8.69% of Type I, in about 33.3% of Type II and in all Type III patients. Overall apical pathosis was observed in 20.54% of the study population with DI. These apical pathologies led to 18 root canal treatments, 24 extractions (nine complicated), two dental implant placements, and one orthodontic treatment in 15 patients. For sure the high number of impacted teeth in the study population effected the treatment strategies. These results may explain why studies on DI are so relevant to dentists. The studies show that significant rate of patients with Type III and Type II DI require dental treatment. In the literature, many successful treatment reports were published even for Type III cases of DI (Schwartz & Schindler, 1996; Kumar et al., 2014; Ceyhanli et al., 2014; Vier-Pelisser et al., 2012). In cases with long-term follow-up, the treatment protocol was MTA plug at the apical end and lateral condensation or warm gutta-percha techniques for obturation of the root canals were likely to be preferred (Pérez-Alfayate, Mercadé & Vera, 2020; Zubizarreta-Macho et al., 2019; Alessandro et al., 2018). In a case report by Ceyhanli et al. (2014), the absence of infection in the main root canal by CBCT was confirmed, and the apical lesion only healed by treatment of the invagination canal. The three-dimensionality of the CBCT images facilitated this observation.

The authors must declare that limitations the study which includes not having the chance of investigating full mouth CBCT images of all patients and could not clinically follow up to see the consequences of treatments of DI’s.

The importance of radiologic and clinical correlation in diagnosis and treatment protocol of DI’s is clear. Thus, despite the limitations of CBCT such as using ionizing radiation and relatively high doses of radiation compared to those used in periapical and panoramic radiographs (Ordinola-Zapata et al., 2017), beyond doubt the CBCT imaging is superior to that of the panoramic radiographs in detecting DI. This is an important point to mention that due to the probability of high incidence of apical pathologies that may develop in the presence of DI, CBCT confirmation of suspected cases should be revised regardless of the patients’ age. Conventional two-dimensional radiographic imaging has been used as the primary diagnostic tool however has some limitations, such as distortion projection errors, angulation errors, and overlapping of anatomical structures which are projected on a two-dimensional plane. CBCT, which is free of any overlapping, would provide accurate information (Mallineni, Anthonappa & King, 2016).

In this study, besides the prevalence and type of DI, the presence of apical pathosis and prophylactic treatments were specially examined. Cone-beam computed tomography imaging can be effective in the detection of dental anomalies such as dens invaginatus and planning for root canal therapy and surgical treatments. Prophylactic interventions might be possible to prevent apical pathosis with the data obtained from cone-beam computed tomography images.

In addition to the frequent occurrence of DI in anterior teeth, radical surgical treatments, that had to be done especially when not timely diagnosed, can lead to unwanted functional and aesthetic consequences. As a matter of fact, the anatomy must be well understood when treating dental anomalies and choosing the treatment to be applied. Therefore, it is crucial that the dentists should treat these cases by examining the CBCT images and, as such, use different methodologies whenever necessary (Oda et al., 2016).

## Conclusions

Dental anomalies should be carefully investigated and timely diagnosed. CBCT imaging could be highly effective for the investigation of the complex anatomy inherent to cases of DI. Not only have CBCT images contributed to the treatment of teeth affected by DI, but they have also significantly contributed to the detection of the affected teeth, frequency of DI, and even the possible local etiological factors affecting DI evaluation. Among the wide varieties of treatment options, predictable and successful outcomes are the consequences of sensitive evaluation and application of the dental professional. Future studies on the etiology might bring new insights to the treatment options.

##  Supplemental Information

10.7717/peerj.14450/supp-1 Supplemental Information 1 The distribution of the prevalence, characteristics, and types of dens invaginatus cases examined The prevalence, characteristics, and types of dens invaginatus (DI).

## Additional Information and Declarations

### Data Availability

The following information was supplied regarding data availability: The raw data is available in the Supplementary Files.
